# What is the performance of the Aspartate Aminotransferase to platelets Ratio Index in patients With Chronic Liver Disease?

**DOI:** 10.5812/kowsar.1735143X.798

**Published:** 2011-12-20

**Authors:** Jean Pierre Zarski

**Affiliations:** 1 Department of Hepatology and Gastroenterology, Digi-DUne Research Center, Grenoble University Hospital, Medical officer of the pole, Grenoble, France

**Keywords:** Aspartate Aminotransferase, End Stage Liver Disease

Dear Editor,

At present, liver biopsy is still the standard in assessing liver fibrosis, especially in patients with chronic viral hepatitis. However, biopsy is invasive and carries the risk of serious complications. In addition, the accuracy of liver biopsy is limited as a result of intra- and interobserver variability and sampling error. Several noninvasive direct and indirect serum markers (such as Fibrotest®, Fibrometer®, and Hepascore®) have been developed and proposed for the noninvasive prediction of significant fibrosis and cirrhosis, especially in patients with chronic hepatitis C [1]. These methods have been validated in naïve patients with chronic hepatitis C but have been proposed for the diagnosis of liver fibrosis in chronic hepatitis B, alcohol cirrhosis, and NASH.

Another index is the aspartate aminotransferase to platelet radio index (APRI), which has been reported to identify patients with hepatic fibrosis and cirrhosis [2]. The retrospective study by Yilmaz et al. [3] assesses the diagnostic ability of the APRI with 207 patients with chronic hepatitis B, 108 with chronic hepatitis C, and 140 with NAFLD. Receiving operating characteristics (ROC) curves were used. The results showed that the APRI was significantly associated with fibrosis scores in patients with chronic hepatitis C and NAFLD but not in those with chronic hepatitis B. In patients with chronic hepatitis C, the APRI showed a sensitivity of 72.7%, a specificity of 62.4% for diagnosis of fibrosis, and an AUROC of 0.582. In the NAFLD group, the AUROC was greater than or equal to 0.627, but in patients with chronic hepatitis B, the AUROC was less than or equal to 0.541.

These results are different from those observed in a recent metaanalysis published by Lin et al. [4]. In this metaanalysis, 40 studies with a total of 8,739 patients were reviewed. The AUROCs of the APRI for diagnosing significant fibrosis, severe fibrosis, and cirrhosis were 0.77, 0.80, and 0.83, respectively. The differences observed between Lin et al.'s metaanalysis [4] and this study [3] are probably due to spectrum bias and a lack of potency. In addition, the variations observed between the AUROCs of chronic hepatitis C and B patients for the different blood tests have been previously published for all scores and could be due to a different distribution of fibrosis in the two liver diseases, especially the greater perisinusoidal fibrosis usually observed with chronic hepatitis C than with chronic hepatitis B. In a recent study that compared Fibrotest®, Fibrometer®, and Hepascore®, we observed that the cutoffs could be different and usually lower in patients with chronic hepatitis B than in patients with chronic hepatitis C. This study seems to confirm that we cannot use blood with the main cutoff in both types of viral chronic hepatitis. This recent study suggest that the APRI can identify hepatitis C and NAFLD-related fibrosis with a moderate degree of accuracy, although not as well as Fibrotest®, Fibrotest® and Hepascore®. However, this blood test is cheap and easy to perform and may decrease the need for taking liver-biopsy specimens among chronic hepatitis C and NAFLD patients.
